# The hidden emotional labour behind ensuring the social value of research: Experiences of frontline health policy and systems researchers based in Kenya during COVID-19

**DOI:** 10.1371/journal.pgph.0002116

**Published:** 2023-08-29

**Authors:** Jacinta Nzinga, Jacquie Oliwa, Dorothy Oluoch, Joyline Jepkosgei, Daniel Mbuthia, Mwanamvua Boga, Peris Musitia, Muthoni Ogola, Naomi Muinga, Kui Muraya, Alex Hinga, Dorcas Kamuya, Maureen Kelley, Sassy Molyneux

**Affiliations:** 1 Health Systems and Research Ethics Department, Kenya Medical Research Institute (KEMRI) Wellcome Trust Research Programme, Nairobi, Kenya; 2 Population Council, Nairobi, Kenya; 3 Nuffield Department of Clinical Medicine, Centre for Tropical Medicine and Global Health, University of Oxford, Oxford, United Kingdom; 4 Nuffield Department of Population Health, Wellcome Centre for Ethics & Humanities, University of Oxford, Oxford, United Kingdom; ESIC Medical College & PGIMSR, INDIA

## Abstract

Health policy and systems research (HPSR) is a multi-disciplinary, largely applied field of research aimed at understanding and strengthening the performance of health systems, often with an emphasis on power, policy and equity. The value of *embedded and participatory* HPSR specifically in facilitating the collection of rich data that is relevant to addressing real-world challenges is increasingly recognised. However, the potential contributions and challenges of HPSR in the context of shocks and crises are not well documented, with a particular gap in the literature being the experiences and coping strategies of the HPSR researchers who are embedded in health systems in resource constrained settings. In this paper, we draw on two sets of group discussions held among a group of approximately 15 HPSR researchers based in Nairobi, Kenya, who were conducting a range of embedded HPSR studies throughout the COVID-19 pandemic. The researchers, including many of the authors, were employed by the KEMRI-Wellcome Trust Research Programme (KWTRP), which is a long-standing multi-disciplinary partnership between the Kenya Medical Research Institute and the Wellcome Trust with a central goal of contributing to national and international health policy and practice. We share our findings in relation to three inter-related themes: 1) Ensuring the continued social value of our HPSR work in the face of changing priorities; 2) Responding to shifting ethical procedures and processes at institutional and national levels; and 3) Protecting our own and front-line colleagues’ well-being, including clinical colleagues. Our experiences highlight that in navigating research work and responsibilities to colleagues, patients and participants through the pandemic, many embedded HPSR staff faced difficult emotional and ethical challenges, including heightened forms of moral distress, which may have been better prevented and supported. We draw on our findings and the wider literature to discuss considerations for funders and research leads with an eye to strengthening support for embedded HPSR staff, not only in crises such as the on-going COVID-19 pandemic, but also more generally.

## Introduction

A central refrain motivating research throughout the immense challenges of working in a global pandemic has been the appeal to the critical social value of rigorous, ethical research. Given the scale and nature of the COVID-19 pandemic, the urgency of conducting high quality, timely research, including health policy and systems research (HPSR), was clear from the outset [1,2].

HPSR is a growing, multi-disciplinary and largely applied field of research aimed at understanding and strengthening the performance of health systems, often with an emphasis on power, policy and equity [3,4]. Over the last ten years, *embedded* HPSR has been highlighted as a relatively innovative approach to conducting HPSR [5,6]. Embedded HPSR involves researchers working inside or alongside a host organisation, facilitating the understanding of complex, sometimes less visible, context-specific issues and influences on health system functioning [7]. The approach can ensure greater relevance and impact in policy priority-setting and decision-making [7]. Embedded forms of HPSR can assist in understanding how shocks to the health system such as pandemics, epidemics and dramatic climatic events (earthquakes, flooding etc) impact on health systems, and how health systems respond and transform in such periods of particular stress; shocks that are typically additional to more chronic, continuous stresses such as resource constraints [1].

Learning from past epidemics, many HPSR researchers joined others right from the outset of the COVID-19 pandemic to call for responses, including research, to consider the wider health and social system impacts in the short, medium and long term, and for social justice concerns to be taken seriously [1,8,9]. Gilson et al [1], for example, in their collective advocacy piece for HPSR, highlighted the potentially critical role of health systems in responding to COVID-19 and the need to (re-) invest in these systems through the state. They proposed a structured research agenda, including a set of emerging HPSR themes and topics and ideas about how the HPSR community might do research differently [1].

Gilson et al outlined some of the valuable HPSR work conducted in Low Middle Income Countries (LMICs) in the early months of the pandemic, and many others have highlighted further invaluable work since then. However, a gap in the literature to date is a documentation of the experiences and coping strategies of the HPSR researchers who were embedded in resource constrained health systems during COVID-19. This is important because COVID-19 not only introduced shocks and stresses to health systems, but also to the HPSR staff conducting those studies, and the health system staff, managers and policy makers they work alongside [10].

Although there are debates in the field of HPSR on best practice, it is recognised that establishment of relationships that support knowledge sharing are key, and that there are ethical challenges related to complex interactions and relationships between researchers, community members, health providers and managers [11,12]. Where roles are blurred between researcher and participant, there can be particular challenges related to maintaining trusting relationships across multiple actors, in managing expectations appropriately, and in consent processes [13]. Rigid ethics review and research funder systems can exacerbate challenges, with some ‘solutions’ to dilemmas (for example implementing continuous consent processes) leading to new issues and complications [14,15]. How to ensure meaningful, mutually beneficial collaboration and maintain the unique value of embedded HPSR whilst navigating a serious public health crisis remains underexplored.

An ethical issue given inadequate attention in ethics guidance and literature, but likely to be a particular concern for HPSR researchers embedded in health systems in LMICs during crises, is emotional distress of frontline staff, linked to the unspoken emotional labour and compromises involved in building and maintaining relationships. Interactions in resource constrained contexts can challenge researchers’ values, competencies, priorities and well-being even outside of a crisis like the pandemic [16,17]. However, research ethics guidance has tended to focus on the protection of study participants, ensuring their confidentiality and non-exploitation in participation, the priorities, concerns, vulnerabilities, and agency of frontline research staff in conducting research in LMICs. There is also growing interest in ethics guidance in fairness between institutions (especially in the Global North and South) in who benefits from global health research. Research that has been conducted on the challenges and emotional distress faced by frontline research staff [18–22] has to date focused more on ‘fieldworkers’ working in homes and communities, than on researchers embedded in health systems [22,23].

We need to learn from decades of careful work on participant protection, more recent emphasis on strengthening equity in global health research and from emerging initiatives aimed at understanding vulnerabilities and risks for frontline staff, on how we can work towards protection and support for research staff as a central concern in research practice. In this paper we share experiences of members of the KEMRI-Wellcome Trust Research programme (KWTRP) conducting embedded HPSR research through the COVID-19 pandemic. Documenting the shocks and stressors the staff faced, the coping mechanisms adopted, and the implications for the conduct of COVID-19 specific and broader HPSR, we offer insights into support processes for research staff in the face of future shocks and more generally. This is essential to producing high quality research with immediate and longer-term social value.

### Study setting and methods

This paper draws on qualitative methods to present the collective experiences a large team of HPSR researchers at the KWTRP, including most of the authors, most of whom have been conducting HPSR work within the programme for over 10 years.

#### HPSR work in KWTRP

Health systems and policy work within KWTRP started in the early 2000s before the field of HPSR was well-defined. It has grown hugely over the last twenty years, in capacity, number and multi-disciplinarity of researchers involved, and range and scale of studies conducted. Current HPSR studies include health financing and health economics work, health product and technology studies, research on health systems governance and leadership and work on human resources for health and health service delivery.

**Fig 1:**
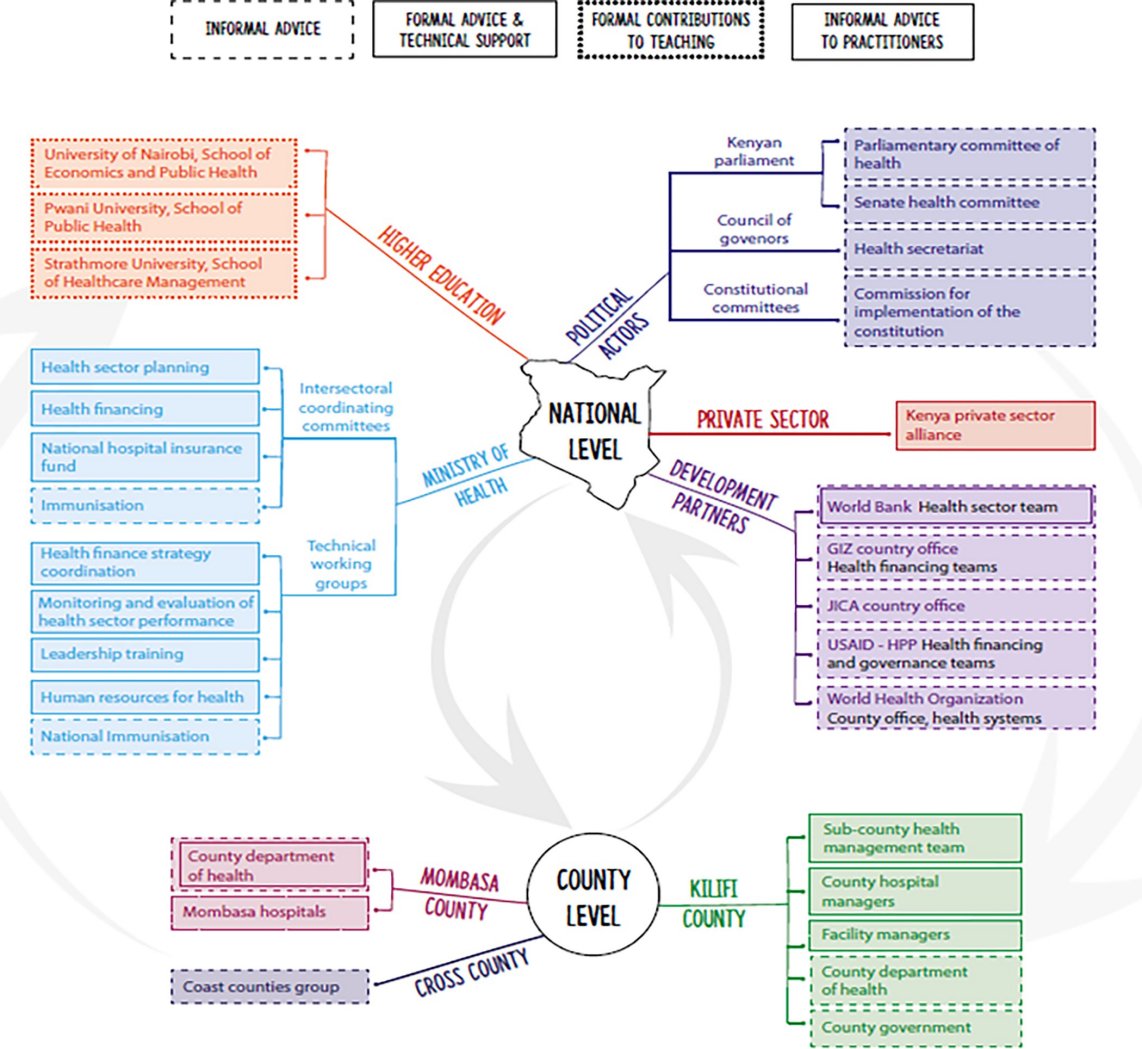
Mapping KWTRP’s researcher’s embeddedness within the Kenyan Health System (borrowed from the RESYST (Resilient and Responsive Health System Consortium) https://resyst.lshtm.ac.uk/).

All of these HSPR areas have an explicit and deliberate focus on policy engagement, and all seek to be responsive to stakeholder needs and priorities, particularly those of Ministry of Health, patients and communities. Although studies fall across a wide spectrum in terms of levels and types of embeddedness in the health system, the cornerstone of researchers’ efforts is to design and conduct locally and nationally engaged and responsive research. Studies typically involve close collaboration with stakeholders spread across the system including policy makers at national and county level, health managers of varying seniority, training institutions, professional associations, frontline health professionals, community members and advocacy groups (as illustrated in Fig 1). For example, HPSR researchers sit on national intersectoral coordinating committees and technical working groups, interact with county level stakeholders, teach in national universities, collaborate with development partners, are members of private sector initiatives and with an expanding network of locally elected community representatives [24,25].

The embedded approach taken by KWTRP HPSR has been made possible by leveraging on strong and often long-term collaborations and facilitative relationships with stakeholders. In particular, two long-term embedded research approaches have served as ‘platforms’ for a series of HPSR studies over time. One is the Kenyan Clinical Information Network (CIN) which facilitates regular practitioner problem solving for hospital paediatric care, where peer learning and accountability is encouraged around the use of hospital data to inform paediatric care decision making [26,27]. The other is the Kilifi Learning Site where joint reflective engagements between researchers and managers are aimed at deepening shared understanding of governance and leadership experiences to strengthen everyday health system resilience and improve system responsiveness to community priorities and concerns [28]. These platforms are supplemented by a robust institutional public engagement programme which includes policy, community, school and university engagement platforms [29].

## Methods

The data shared in this paper are drawn primarily from monthly HPSR research staff team meetings held online throughout covid-19, and a series of additional specifically organised smaller in-depth group discussions on the practical and ethical dilemmas raised through the monthly meetings.

### Monthly HPSR meetings

As part of the hard lockdown implemented by the Kenyan government with the first COVID-19 case in March 2020, almost all non-COVID-19 studies, including HPSR studies, were required to close or ‘hibernate’. It took several weeks for HPSR researchers across KWTRP to re-orientate themselves and -re-establish previous meetings, but online. Approximately 15–20 HPSR researchers from KWTRP joined these monthly social science reflection meetings from May2020-October 2021, with most meetings lasting 45 to 60 minutes. Discussions focused on staff and study updates, changes to existing research activities, planned new studies (primarily COVID-19 related given the context), and stakeholder engagement strategies.

Over time, personal, professional, and ethical challenges of conducting research during the COVID-19 pandemic were discussed, and responsibilities to health system collaborators in such a stressful period debated. It became clear that we needed to discuss the practical and ethical challenges we were experiencing in more detail, and so organised small group discussions for those leading and conducting HPSR studies who were experiencing greatest challenges.

### Group discussions

We organised a series of five, two hourly sessions attended by 10–15 people per session, to discuss practical and ethical issues being experienced in more detail, incorporating a colleague and external ethics advisor (MK) to support us in this process. As will be discussed below, these sessions became a valued space to raise and share issues. Those attending the discussions (co-authors) were working on a range of studies as summarised in *Table 1*, which also illustrates the varied ways these studies were embedded in into national decision-making and implementation structures and systems.

**Table 1:** Summary of some of the KWTRP embedded HPSR projects undertaken over COVID period.

	Research Project	Nature of embeddedness
1	The African Health Observatory Platform on Health Systems and Policies (AHOP)	• Co-production and synthesis of evidence with policy makers • Research team part of the TWGs, ICCs and secondment to the MOH
2	Role of nurses and nursing teams in improving routine service delivery and during pandemics (part of CIN)	• Co-development of research questions with policy makers • Hospital ethnographies involving embedded observation work
3	Co-design of interventions for neonatal clinical team • a small and sick newborn clinical audit tool and implementation guide. • a newborn monitoring chart, online launch and implementation evaluation • a participatory communication skills and emotional competence course for nurses	• Co-design of interventions with frontline health workers to strengthen neonatal care • Agree interventions, implement and evaluate
4	Analysing global and national policies for strengthening critical care services in the context of the global COVID-19 pandemic	• Co-development of research questions with policy makers • Research team part of TWG and steering committees for emergency and critical care
5	Exploring the potential for learning and using parent experiences of preterm birth to improve in-patient neonatal care in Kenya.	• Co-design of a nurses communications training. • Involve nurse Trainer of Trainers (ToTs) in the implementation of the developed course training
6	Examining men’s engagement in Child Health in Urban Informal Settlements of Nairobi	• Qualitative exploration of individual and system level barriers and facilitators of men engagement in child healthcare • Co-development of a set of behaviour change interventions that can be implemented at community level and translated to policy

These discussions/reflection sessions covered the following inter-related topics which had been raised in the monthly meetings:

- Dilemmas of progressing work without adding pressure on busy colleagues
- Shifted priorities for stakeholders–‘distorted’ asks and use of ‘evidence’
- Witnessing complex needs of participants and not feeling able to respond in a sustainable way, ‘within the system’
- Experiences of obtaining ethical approvals
- Experiences of emotional ‘burn out’ especially among clinical HPSR staff

Across these topics we sought to distinguish between more practical and ethical issues. We discussed opportunities, challenges and tensions for protecting the well-being of both the researchers and our health system participants during this and future crisis periods and balancing those needs against continuing to actively engage stakeholders in order to conduct high quality, responsive, research.

### Analysis

Detailed minutes were kept of the monthly meetings, and we sought consent from all researchers who attended the reflective meetings to record the proceedings, redacting only the small details of the recordings that participants felt should not be shared.

JN and SM re-listened to the recordings between each group discussion, making very detailed minutes from them to plan and feed into future group meetings. Using this manual, iterative approach in word documents, they reviewed issues being raised about participants’ immediate needs, coping strategies (and any associated dilemmas), and recommendations for future. These issues were clustered into the topics listed in the previous section, seeking to distinguish between more practical and ethical issues where possible. These issues were then grouped into 3 main thematic areas around which the findings are organised: (i) promoting the social value of research versus responding to immediate needs; (ii) shifting ethics procedures and processes to accommodate research needs during the pandemic; and (iii) staff well-being and emotional challenges.

## Results

The findings discussed under the three main themes are primarily ethical, with the practical challenges and staff responses summarised in Box 1. However as will be seen, practical issues had important implications for the quality of research and therefore the potential social value of the research (theme 1), and for staff well-being (theme 2). The practical and ethical challenges and responses are therefore often interwoven.

**Box 1. Practical challenges and strategies for balancing rigorous research methods within pandemic needs and constraints**

**Overall**:

○ Stakeholder priorities shifted rapidly and prioritised rapid covid-19 related data
○ We responded to shifts and requests through drawing on an existing platform of relationships and interactions, pivoting existing work to add new foci, providing regular research syntheses and working longer and more flexible hours

**For the shift to virtual data collection and digital engagements**:

○ Keeping the camera on to ensure active engagement
○ Conducting interviews in sections, or shorter interviews–focusing only on the priority issues and being strategic with questioning and reducing the length of the guide. Also having the questions less formalised and structured. Sometimes compensating for this with increasing the number of participants
○ Requesting people in authority to call for meetings in order to gain the interest of their colleagues e.g., Asking the head of directorates in the MOH to host meetings improved attendance and buy in
○ Working flexibly and shifting work hours to fit with others, including working in the evenings and early mornings. One interview was even conducted at 5.30am by request of a participant. Another challenge was the concern about adding burdens to participants and intruding even into their evening/personal time

**Across all of our interactions and engagements**:

- Balancing shorter interactions with demonstrating empathy with our research participants about shared concerns and challenges and showing how much we value their insights and experiences
- Being open and upfront about the challenges we were facing as researchers and the pressure of the funder timelines
- Minimising overburdening people with research through for example establishing and maintaining a shared calendar across researchers engaging with health managers and workers. But even this felt sometimes felt like we were pushing boundaries researchers’ and participants’ boundaries
- Making trade-offs and recognising that some information we wanted (for example on use of covid-19 funds) was simply too sensitive to collect through interviews.
- Sharing dilemmas and concerns and agreeing responses in a safe space

### A. Social value of research vs responding to immediate needs

A set of dilemmas that emerged early in the pandemic was if and how to respond to a shift in what research was being prioritised by health system collaborators, away from existing research. Dilemmas included if and how to switch to COVID-19 specific HPSR work or add an HPSR lens, how to ensure social value of research that depends on stakeholder interest and engagement in such a difficult environment, and potential risks and harms to staff, services and (future) research.

### A shift in stakeholder priorities and concerns

Although initially there was hope that the pandemic would soon be controlled and that previously valued non-COVID-19 specific research would quickly resume, as the pandemic began to intensify, stakeholder priorities began to shift almost exclusively towards COVID-19 related research and engagement. This was particularly observed among policy makers at national and sub-national levels. For example, most of the African Health Observatory Platform on Health Systems and Policies (AHOP) work (A regional collaborative initiative that supports and promotes the transfer of evidence and experience between countries to foster action. https://ahop.aho.afro.who.int/) (Table 1), focused on co-producing and synthesising research evidence, requires repeated engagement and buy-in from policy makers. Until COVID-19, AHOP work had been focusing on routine evidence generation to guide health system investment decision-making. However, with COVID-19, much of this work had to be put on hold as stakeholders no longer considered it a priority.

Indeed, almost all studies involving mid-level managers and frontline health workers were initially put on hold: many mid-level managers had to reorient their systems and services to respond to COVID-19. Additionally, managers with clinical training were having to assist directly in providing care, including to alleviate workload for frontline health workers who were covering for colleagues off work or deployed to assist in COVID-19 centres. Shifting work demands for frontline staff coupled with increasing fear of contracting COVID19 amongst many meant that most health worker related research was difficult to undertake and risked adding burdens to an already over-stretched and anxious staff. At the same time, some HPSR researchers were ‘blasted’ by health system collaborators for ‘knowing the problem (of COVID19) but doing nothing to solve them’, raising concerns among HPSR researchers about damaging existing relationships with colleagues essential for current and future research.

### A corresponding shift in research priorities and plans

HPSR researchers could see potential learning about the role of health systems in responding to pandemics, and the impact of COVID-19 on health services, systems, and patients. HPSR had the potential to contribute to the national (and international) COVID-19 response, and to positive transformation of health systems to respond timely to the current and future pandemics and shocks. Some research teams therefore re-strategized their research to include a COVID-19 research element. For example, the pandemic provided AHOP with a policy window to engage policy makers with priority evidence for real-time decision making on the COVID-19 response and containment measures [30]. Thus, several research teams submitted new COVID-19 related proposals or amendments to existing proposals (adding a COVID-19 lens) for science and ethics approval.

Some HPSR research teams felt too overstretched or ill-placed to conduct new HPSR with meaningful social value within required timelines and given the difficulties of conducting research primarily online. For example, those working on the ‘male engagement study’ considered building on past relationships and data to contact participants to understand COVID-19 related concerns and priorities of vulnerable groups; but were concerned that conducting the research would add burdens and potentially physical risks to already vulnerable families’ participants without contributing adequate social value through impacting on policy and practice as intended. These researchers, and others, decided to focus instead on analysis and write up of existing data, and on developing policy/issue briefs and publications to support the COVID-19 response [see for example Policy Briefs–COVID-19 (kemri-wellcome.org) and https://extranet.who.int/iris/restricted/bitstream/handle/10665/350527/9789290234593-eng.pdf, https://extranet.who.int/iris/restricted/bitstream/handle/10665/350529/9789290234586-eng.pdf].

Some research that was planned was hampered by ethics approval requirements (as outlined below).

### Pushing boundaries of established relationships to get research done

For studies that were approved, there were difficult decisions about how to conduct the research without adding to the daily burdens of staff and health managers during a difficult and uncertain time. For example, to ensure we did not disrupt people’s call of duty in responding to the pandemic, we organized meetings and interviews outside work hours. However, sometimes there was no response to invitations, and when there was a positive response, there were new dilemmas about taking up personal time, and (for in-person meetings) about potential risk of exposure to COVID-19. While virtual engagements provided greater flexibility and allowed a wider range of participants, researchers had to leverage existing relationships and social capital to make these engagements work. We sometimes felt we were ‘pushing the boundaries’ of those relationships to keep research going.

Concerns emerged over the quality of the data. With limited physical interactions allowed by our institution, and our own concerns about disrupting health providers and even service delivery, most data were collected virtually. However, over time attendance of online meetings dropped, and discussions during meetings with our stakeholders reduced, making it difficult to assess engagement. Furthermore, the informal ‘off the record’ discussions so invaluable in offering rich and sometimes sensitive data pre-COVID-19, were no longer possible, particularly with participants we did not have pre-existing relationships with. It was difficult to cultivate rapport online.

### Responding to immediate non-research needs

Due to the long-lasting relationships that most of our research teams had with health system stakeholders (typically as research participants or collaborators), researchers were often asked to assist with protective equipment, vaccines, other supplies and training. Many of us were keen to assist at an exceptionally difficult time, but also had to deliver on our core research roles as outlined above and were constrained by institutional rules and restrictions. Those with clinical training were able to assist in clinical care, and at an institutional level colleagues contributed centrally to the national COVID-19 testing service and to synthesising data and advice on the national COVID-19 response [31]. Otherwise, we helped in small ways where feasible at an individual or project level. For example, we were able to re-pivot an on-going communication and management of emotions course being evaluated in one study (Table 1) to offer support to health facility managers experiencing significant distress. However, and as described in our previous work, initiatives inevitably felt piecemeal and inadequate given the scale of the challenges faced [32].

### B. Shifting ethics procedures and processes

**Fig 2:**
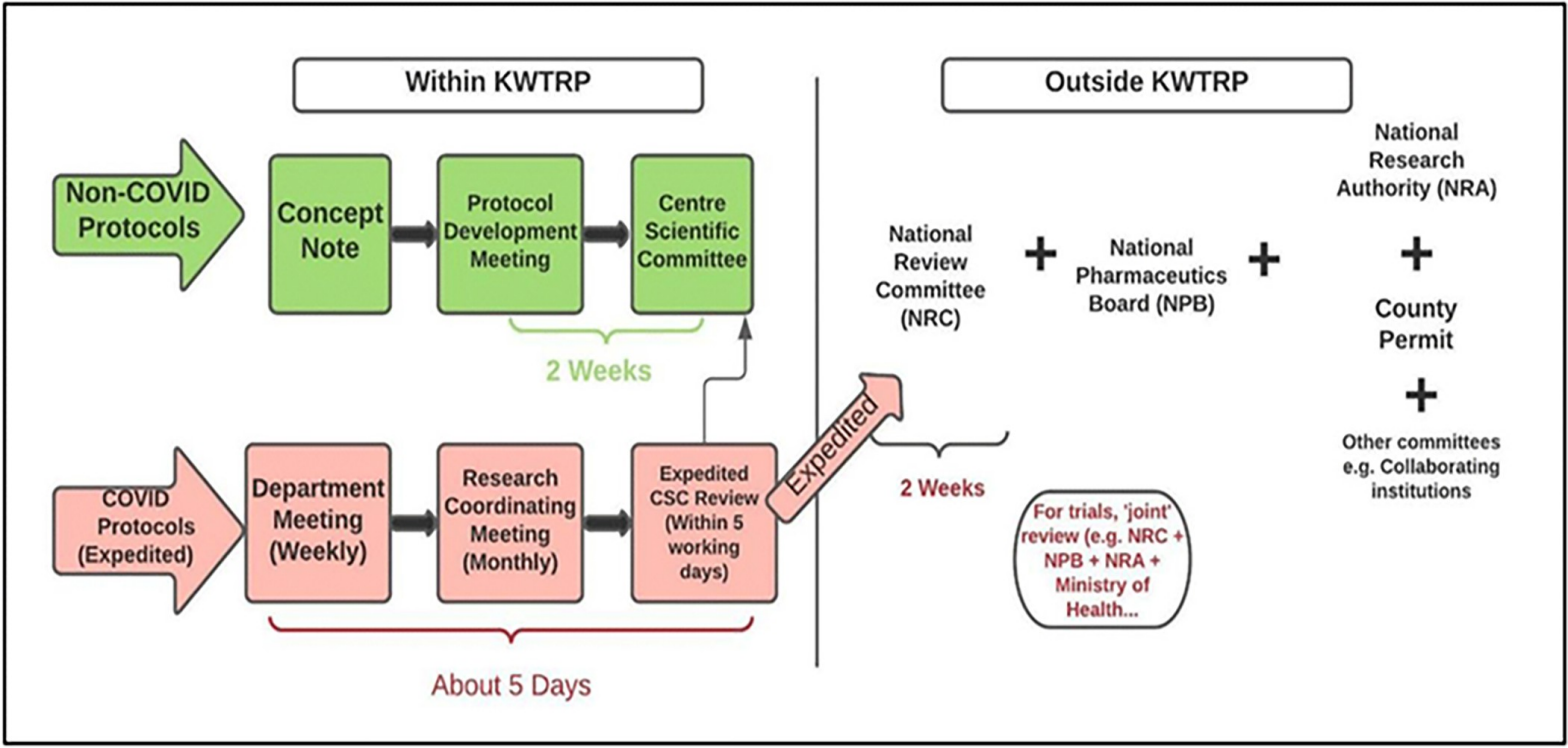
Research ethics process in KEMRI-Wellcome trust research programme.

In Kenya, changes were made to the ethics review systems at the institutional (KWTRP) and national review levels to respond to the specific ethical issues raised by the COVID-19 pandemic. New guidance was developed for example for informed consent and participant recruitment and safety, and new review structures and processes introduced [30]. COVID-19 protocols were to be prioritised with a targeted turnaround time for initial review at the national review level to 14 days (Fig 2). *A*s documented elsewhere [30], between April 2020 and April 2021, 66 protocols were reviewed at KWTRP, of which 30 were COVID-19 related new protocols (n = 10) and amendments (n = 20). HPSR researchers experienced both opportunities and challenges in achieving timely review of COVID-19 proposals, and in implementing new guidance post approval, as outlined next.

### Long review processes for research

Most COVID-19 proposals were submitted in the first six months of the pandemic hitting Kenya, led by principal investigators in the Health Systems and Research Ethics (HSRE) and Epidemiology and Demography (EDD) departments (although much of research that was submitted from all departments was multi-disciplinary). Amendments to existing HPSR proposals were relatively straightforward, essentially requesting for interviews (and associated consent processes) to be conducted online rather than in person. All COVID-19 specific new HPSR proposals were aimed at contributing to the national COVID-19 response in the short or long term.

Although some of the proposals were reviewed relatively quickly, with approval received within three months of submission, some took significantly longer, particularly those that were part of larger multi-disciplinary programmes of research with a clinical trial component. Changes in the review process unintentionally increased the practical complexity of obtaining study approval, in part due to increased government involvement in regulating ethics review processes. This involvement may have been in response to fears (heightened by media coverage of inappropriate comments by foreign researchers) of exploitative research being conducted by international research bodies in Africa. Additional layers of approval (e.g., NACOSTI (National Commission for Science, Technology and Innovation), county, hospitals, KEMRI occupational safety), combined with the sheer volume of proposals, delayed timelines for data collection even for HPSR research being requested by national stakeholders to support with the national/local response. HPSR researchers expressed some frustration with apparent delays to research they felt was potentially valuable, particularly given that questions asked were primarily administrative rather than ethical.

### Understanding and implementing new ethics guidance

With new occupational safety measures in place, almost all research had to be conducted using virtual platforms but–at least initially—without clear understanding on what compensation and benefits participants should be given (ethical issue), and how it should be given (a more practical concern). To illustrate the challenge: for embedded research pre COVID-19, in-person HPSR research interviews and meetings with health managers and health workers were often held during work hours. For short meetings of up to an hour or so, to minimise introducing inequities and tensions between staff, these were held in the workplace at a time of convenience for the participant and are considered part of daily work and not financially compensated (as outlined in consent forms).

When meetings shifted online, and staff requested to meet out of hours because they were too busy at work, we were initially unsure if and how to compensate for their time and for the data costs involved. For longer meetings often involving more staff, before COVID-19 these were typically organised in a central place with a need to travel to that place. Participants had travel costs compensated, were given an out of station allowance set by the government, and meals were covered (and accommodation for meetings of more than one day). When meetings shifted online, it was unclear how ‘out-of-station allowances’ could and should work and whether any such allowances would amount to ‘inducement’. Although these dilemmas were relatively easily resolved when shared across the HPSR team (approaches to compensating participants for their time were agreed), individual researchers were initially unclear on how to proceed, leading to different practices between different teams, to sustain relationships crucial to research over time.

With regards to consent processes, these were often felt to be easier to administer in person than online, where it can be difficult for each party to see and respond to non-verbal communication. Given that consent processes must happen at the outset of research interactions, and are ideally revisited throughout research engagements, this had implications for the entire online discussion and the quality of the data collected. Related to this, there was the practical challenge of how to obtain evidence of consent in the absence of physical signature on the consent form: recorded consent on encrypted Dictaphones and sharing of consent forms via email (where possible) was used to address this challenge. However, this process also brought about other procedural and bureaucratic practicalities, given the short time slots to obtain the signatures and busy and stressed participants often feeling the process was undermining rather than supportive of respectful relationships and quality data. We also had to carefully think through how to handle non-response in contexts where many people were overwhelmed by emails and phone calls (such as sending three reminders by email and making one call and if there is no response, assuming a refusal).

A final challenge in implementing guidance was in knowing if and how we could use data collected as part of support processes to contribute to the unfolding local and national COVID-19 response. For example, when interacting and learning about pressures and stresses faced by health system staff at all levels, we wanted to share what we were learning. In the emotional management support activities described above we did not publish but co-designed an issue brief with the nurses concerned, who were keen to have their issues shared with health managers. Our interest in sharing what colleagues and the public were going through was exacerbated by the distress we felt as KWTRP staff of being supported to work (with health and safety precautions, -double vaccine, space in vehicles, psychological support) whereas many of our stakeholders did not enjoy such privileges. Feelings of inequity were particularly stark when we were required to wear PPE (masks, have hand sanitizers etc) and arrive at venues alone in vehicles to interact with participants who arrived in packed public transport.

### C. Staff well-being and coping with emotional distress

The group/reflective sessions we set up amongst HPSR researchers started off as a safe space to discuss the challenges of conducting HPSR during a pandemic and to support one another. In these sessions, researchers described the exhaustion they were experiencing in trying to keep work going. For example, endless phone calls to arrange meetings and interviews, and constant leveraging on social capital and close relationships to keep stakeholders interested whilst appreciating their role in the COVID-19 response. For many researchers this was in addition to difficulties of working from home, including challenges with electricity and internet access, inadequate or overcrowded accommodation, and multiple disruptions and demands from family members.

‘*It was challenging*, *trying to reach people*, *email after email*, *…and trying to call to book appointments and most not wanting to engage virtually’*. *Participant 3*

“*Yeah*, *one of the things which I think worked very well for me to continue collecting data was being flexible and to giving people time to be flexible*. *So*, *for example*, *informing them that everything would be virtual*. *Uhm and if that meant off certain hours*, *normal hours or even weekends*, *so just providing that for them to kind of choose what time works best for them was helpful*. *But* that was *really hard cause all work hours seem to blur into personal life and work seems to take over everything’*. *Participant 4*

At the same time, participants and people in our personal networks–families and friends—associated us with KEMRI-WT and expected us to be able to answer all kinds of questions on COVID-19 and vaccines even at a time when there was huge uncertainty globally on all of these issues.

“*Everybody knows KEMRI as the only Medical Research Institute in the country*, *and so there were instances where you know our participants and even family members felt we had all the knowledge around COVID or how we should be protecting ourselves*, *about vaccines*, *but we weren’t really sharing that knowledge*. *And so*, *it became very difficult for people to look at us past that…we’ve created relationships over a very long period of time*, *and we actually want to maintain those relationships but also without assuming a role that is really not ours”*. *Participant 1*

Underlying these challenges, HPSR colleagues themselves personally suffered loss and ill-health among family members due to COVID-19. They also witnessed deaths of health workers, teachers, mentors and colleagues, all happening in a context of a series of health worker strikes. These tragedies were contributing to fear and feelings of burnout among our staff, including among those at senior levels who were expected to reach out and support other team members.

“*Early months of 2020 during the first wave*, *it was very scary*, *seeing a lot of loss*, *seeing your teachers die especially nurses who are often much older than doctors and since they are in the hospital most of the time*. *In the Newborn Unit we tried to control the numbers*, *but we failed*, *because we don’t have much space and so it was difficult to distance the patients*.*” Participant 7*.

Some of us faced what could be described as forms of ‘moral distress’, which arises where there are moral uncertainties, tensions, conflicts or dilemmas, or when one knows the right thing to do but institutional constraints make it nearly impossible to pursue the right course of action [33,34]. For example, when there was clear information on COVID-19 available to us we wanted to provide this information to colleagues and the general public but were initially prevented from doing so as a result of government efforts to try to minimise misinformation and conflicting information. We would have liked to provide protective equipment to immediate colleagues in the health system in line with our own policies and procedures but to do so would have undermined the institutional ability to adhere to its’ own policies aimed at protecting its staff.

For clinical HPSR researchers, there were additional physical and social stresses, and moral distress, associated with being a clinical researcher on the frontlines of fighting the pandemic. They were anxious about their personal safety from handling suspected COVID-19 cases and the potential to spread COVID-19 to their families. In handling patients, they had to make difficult choices–as did their health worker colleagues—in the face of limited resources and services about which patients should and should not be admitted.

“*We have very crowded wards*, *and there was no personal protective equipment being provided…you had to source for those yourselves*. *So*, *you were constantly worried about getting sick and getting your family sick…that is what I am grappling with and also personal loss as I have lost some relatives*. *And there is an element of apathy…where health workers are now feeling ‘if I die*, *I die’…and that worries me” Participant 3*.

## Discussion

We did not set out to document our regular meetings through the COVID-19 pandemic but had organised these sessions to support one another to conduct embedded HPSR during a crisis. Our experiences highlight several areas to consider in preparation for future crises, but that are also relevant in non-crisis periods.

### Achieving social value of research in a challenging context

High quality, ethical research plays a crucial role in feeding into and evaluating responses in a crisis. While a wide range of important HPSR themes and topics were highlighted by Gilson et al [1] as potentially valuable to the COVID-19 pandemic, we faced challenges in continuing to conduct embedded studies that we felt had important social value in the short and longer term, in some cases because close collaborators had more pressing immediate priorities. As has been noted by others, collaborators often prioritised more rapid data collection and outputs [35,36]. This highlights differences between stakeholders–heightened during a crisis—in what counts as priority and ‘good enough’ data and what activities have social value.

Even in this context, and partly in response to it, we were able to continue to conduct some embedded research and to initiate new studies, including research reported to have had immediate value for policy makers and practitioners. We were also able to set up regular feeding in of policy briefs to decision-makers nationally and at sub-national county level and to share learning more widely (Policy Briefs–COVID-19 (kemri-wellcome.org)). We did this by drawing on an existing platform of relationships and interactions [31], by pivoting existing work to add a new focus and by contributing significant extra work in difficult situations. We recognised that we could not implement all research activities that were requested of us or that we felt might be useful, and that any research we did conduct would have opportunity costs for all of those involved, in terms of taking up people’s time, energy, and resources [37].

For the research that we did conduct, a central challenge was maintaining relationships with stakeholders so central to our research and collection of quality embedded HPSR, even outside of the COVID-19 context. This at a time when many of our health systems colleagues and collaborators were also having to deal with health worker strikes, and we as researchers were also having to negotiate new ethics review processes and international research funding cuts and uncertainties. Data collection in these contexts was inevitably affected, although we learned many invaluable lessons as participant observers within the systems facing and responding to such unprecedented challenges. Overall, we learned the importance of researcher persistence, solidarity and creativity.

### Moral distress among frontline research staff and strategies to understand and respond to this

The emotional challenges that frontline health workers face are increasingly recognized as a major health systems challenge, with negative implications for staff well-being, mental health, team relationships, absenteeism, retention, quality of care and patient outcomes [38]. Part of the work we conducted during the pandemic, yet to be published but shared in issue briefs, documents the emotional stress faced by so many health systems staff as a result of COVID-19 uncertainties and the national response. In health systems struggling with a wide range of chronic, everyday stressors such as resource constraints, constant policy change, and high staff turnover, high levels of reported stress and ‘burnout’ among health workers can be attributed to moral distress or even moral injury [39]. Moral injury can arise where sustained moral distress leads to impaired function or longer-term psychological harm, potentially producing guilt and shame, and in some cases also a sense of betrayal, anger and profound ‘moral disorientation’ [40]. The systems drivers of moral distress and injury highlight the importance of organisational responsibility and response. Our research and experiences in conducting research alongside health systems staff at all levels support others who have argued for the importance of interventions and ways of working that minimize and manage their moral distress [41,42]. These include structural changes and organisational initiatives such as education interventions, facilitated discussions, creation of formative and relational spaces, and multi-disciplinary rounds.

The emotional labour and moral distress faced by frontline research staff is less widely recognised in the literature than that of frontline health workers [43], and there is little information specifically on the experience of those conducting embedded HPSR, especially during crisis. We found that some of HPSR frontline staff faced similar challenges to health workers, particularly the clinically trained and active staff working in facilities. In many ways this is not surprising: frontline research staff are embedded in the same emotionally challenging, stressful contexts as health workers, and in HPSR research there are grey areas and overlaps between research funded frontline staff and locally employed health workers in roles, accountability, and responsibilities. While our institution was alert to and sought to minimise potential physical harms frontline staff face (for example in introducing new safety and risk assessments prior to fieldwork), the emotional demands and ethical dilemmas associated with working at this interface were given less formal attention, beyond referral to counsellors where this was felt to be potentially important for staff. We were therefore often struggling on our own in working out how best to manage dilemmas before we set up the more specific ethics reflection sessions. These experiences highlight the importance of interventions and ways of working that help minimize and manage moral distress for research staff working in health systems and thinking about if and how they might be shared with health system colleagues with whom we interact. Failure to spread any support processes introduced for research staff to health system colleagues they work alongside, risks introducing new inequities at that interface of health and research systems.

### The importance of long-term, flexible funding to support research with social value and frontline staff emotional well-being

Given the above successes, challenges and needs, the importance of *long-term*, *flexible funding* that can be repurposed in times of crisis is clear. We were extremely privileged as a group to have this from our most prominent funders, particularly at a time when other funders were introducing sudden withdrawals of critical funding. The latter had huge implications for research relationships and contributions.

## Conclusion

Our experiences in conducting HPSR during the COVID-19 pandemic underscore the opportunities, challenges and tensions in conducting ethical research with social value, especially in already fragmented, stressed health systems. These challenges are not unique to COVID-19 but are amplified through such crises. We identified new ways of conducting embedded research while seeking to protect our own emotional well-being and that of our research participants. To maintain the responsiveness and co-production at the heart of the HPSR work we conduct, we had to balance responding to stakeholder needs and priorities with keeping an eye on the longer-term knowledge demands post COVID-19; resisting the temptation to shift all research attention only to COVID-19. In these situations, we relied heavily on a platform of relationships we had built with our stakeholders over the years, being careful not to overstep boundaries, and pivoting funds were allowed by flexible sponsors. Our experiences demonstrate that in navigating research work and responsibilities to colleagues, patients and participants, many staff faced difficult emotional and ethical challenges, including heightened forms of moral distress, which may have been better prevented and supported. The importance of flexible funding to respond to crises and of ensuring that interventions and ways of working that minimize and manage moral distress are funded is highlighted.
